# Supplementary material for: Long-Term Efficacy of AAV9-U7snRNA-Mediated Exon 51 Skipping in mdx52 Mice
Source: Mol Ther Methods Clin Dev. 2020 May 4;17:1037–47. doi: 10.1016/j.omtm.2020.04.025 (PMC7240049; doi:10.1016/j.omtm.2020.04.025)
Supplement: Document S1. Figures S1–S5 and Supplemental Materials and Methods [file mmc1.pdf]

OMTM, Volume *17*

Supplemental Information

Long-Term Efficacy of AAV9-U7snRNA-Mediated Exon 51 Skipping in *mdx52* Mice

Philippine Aupy, Faouzi Zarrouki, Quentin Sandro, Cécile Gastaldi, Pierre-Olivier Buclez, Kamel Mamchaoui, Luis Garcia, Cyrille Vaillend, and Aurélie Goyenvalle

Supplemental information includes:

-5 supplementary figures with figure legends

-supplementary methods related to the supplementary figures

SUPPLEMENTARY FIGURES

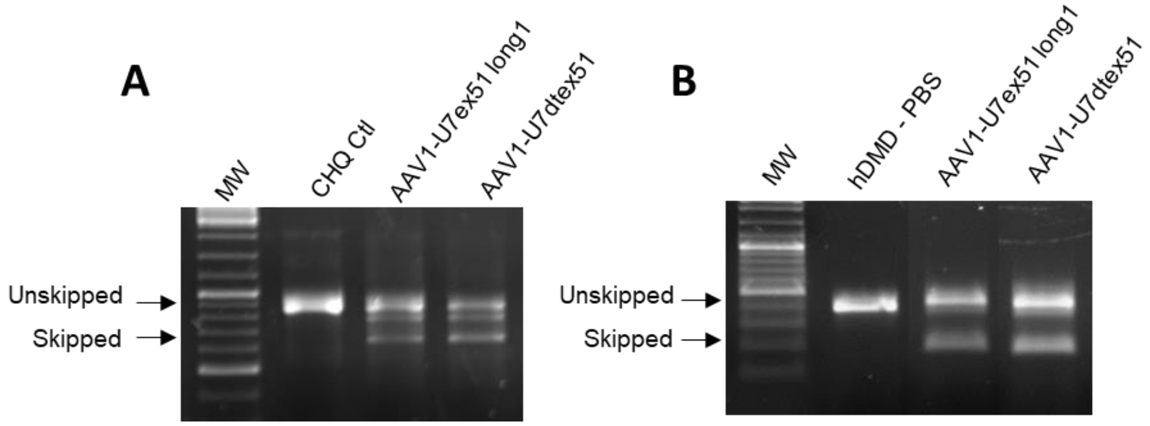

<u>Supplementary Figure 1</u>: **Both U7ex51 constructs efficiently skip the human exon 51 *in vitro* and *in vivo*.** The two different U7snRNA constructs (ex51 long1 and dtex51) were introduced into AAV1 vectors for preliminary evaluation. A) detection of exon 51 skipping in human immortalized skeletal cell line CHQ transduced with 5^{E}+11vg of AAV1 vector encoding the various U7ex51. B) Detection of exon 51 skipping in the TA of hDMD mice injected intramuscularly with 1^{E}+11vg. TA muscles were analyzed 3 weeks after AAV1-U7 injection.

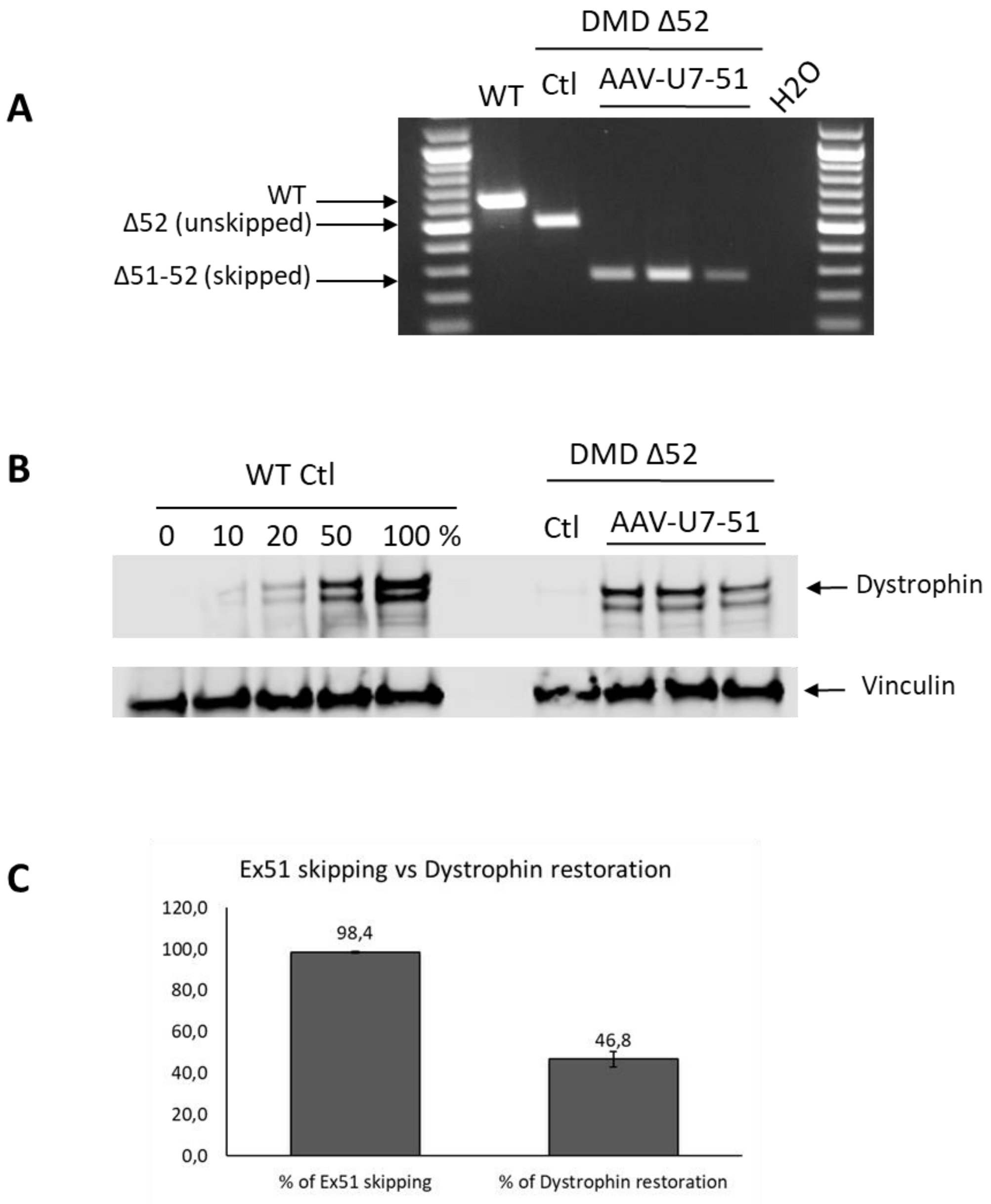

Supplementary Figure 2: Comparison between exon 51 skipping and dystrophin protein restoration *in vitro*. A) Detection of exon 51 skipping in human immortalised DMD Δ52 (KM571) myotubes transduced with AAV1-U7ex51. B) Detection of dystrophin protein in human immortalised DMD Δ52 (KM571) myotubes transduced with AAV1-U7ex51. A standard curve made of WT protein lysate (CHQ myotubes) mixed with DMD Δ52 (KM571) myotubes protein lysate was used to quantify the level of dystrophin restoration. C) Graph presenting the quantification of skipping and dystrophin protein levels. N=3 transduction experiments.

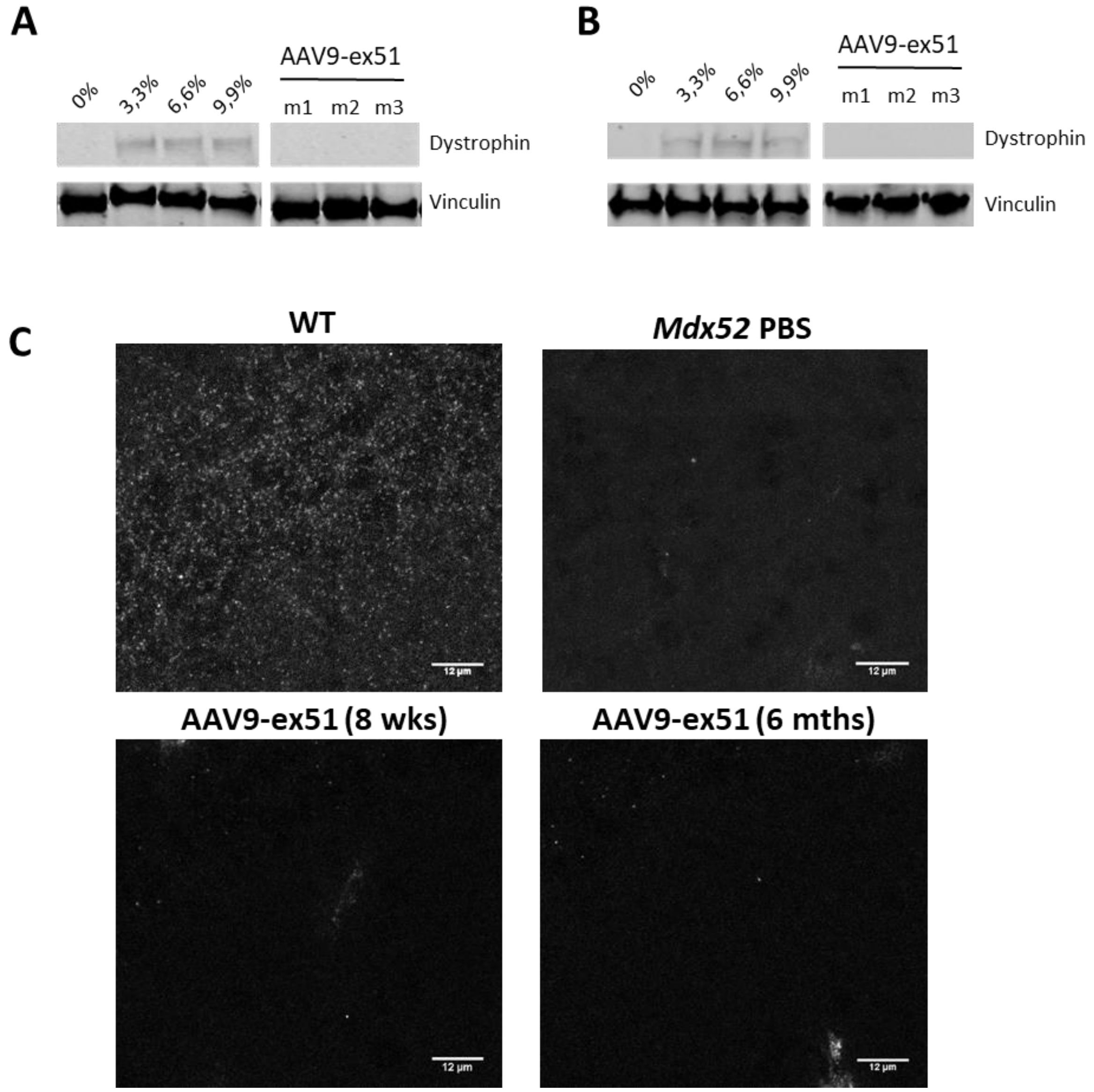

<u>Supplementary Figure 3</u>: **AAV9-ex51 intravenous injection does not restore Dp427 in the brain.** Detection of dystrophin restoration in A) Hippocampus and B) Cortex by western blot compared to control *mdx52* mice (0%) and WT mice. 20µg of total proteins were loaded for all samples, with amounts of WT tissues ranging from 3,3% to 9,9% for the WT control. No dystrophin could be detected in treated mice (n=3 mice). C) Immunostaining of dystrophin in hippocampus (stratum pyramidale) of WT, control *mdx52-PBS and* AAV9-ex51 treated mice, 8 weeks or 6 months after the intravenous injection. Scale bar, 12µm.

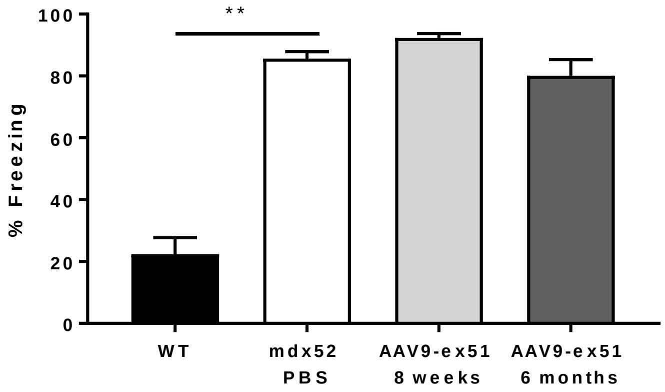

<u>Supplementary Figure 4</u>**: AAV9-ex51 has no effect on *mdx52* freezing response 8 weeks or 6 months post-injection.** Mice were restrained for a brief (10s) period and released to a cage to analyze their fear response. Freezing duration was measured for 5 min. WT mice showed freezing during 20% of the 5-min testing period, while *mdx52* mice expressed a freezing amount of 80%. No improvement was observed after AAV9-ex51 injection. (WT n=5, *mdx52* PBS n=6, AAV9-ex51 8weeks n=6, AAV9-ex51 6months n=3). Results are expressed as means ± SEM. **p<0,01 (Mann-Whitney U tests).

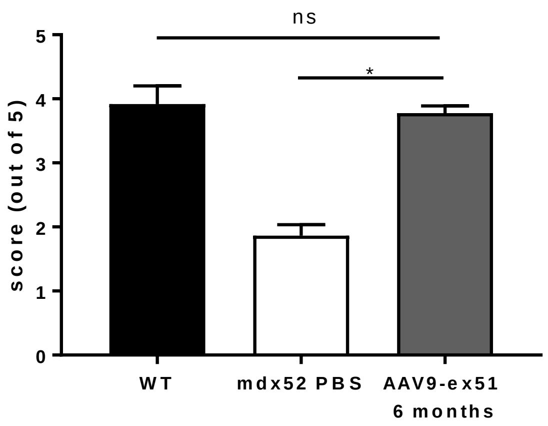

<u>Supplementary figure 5</u>: AAV9-ex51 improves traction capacity in *mdx52* mice. During the wire suspension test, a score was given to determine the mouse capacity to execute tractions. 0= fall directly, 1= grip the wire with forepaws and wait, 2= grip and try traction, 3= successful traction reflex (wire touched by at least one hindpaw), 4= successful traction reflex with all four paws and tail additionally wrapped around the wire, 5= successful traction reflex, tail around the wire and walk along the wire to escape to one of the supports. WT n=4, *mdx52* PBS n=5, AAV9-ex51 n=3. Results are expressed as mean ± SEM. *p<0,05 (Mann-Whitney U tests).

SUPPLEMENTARY MATERIALS AND METHODS

Cells and AAV transduction

Immortalized human skeletal muscle cells derived from a healthy subject (CHQ) and a DMD patient harboring a deletion of exon 52 (ID KM571) in the DMD gene were generated by transduction with human telomerase-expressing and cyclin dependent kinase 4-expressing vectors in the Institute of Myology human cell immortalization platform, as previously described [1]. Cells were cultured in proliferation conditions with Skeletal Muscle Cell Growth Medium (Promocell), supplemented with the skeletal muscle supplement mix (Promocell), 20% fetal bovine serum (Life Technologies), and antibiotics (50 U penicillin and 50 mg/ml streptomycin; Life Technologies). For AAV transduction, 65,000 or 130,000 cells were seeded in 12-well or 6-well culture plates respectively (depending on further analysis required, RNA only or RNA and WB analysis). Two days after seeding, at approximately 80% confluence, medium was replaced with differentiation medium (DMEM with 2% horse serum (GE Healthcare), insulin-transferrin solution (Sigma) and antibiotics) and $1^{E}+12$vg of AAV1-U7ex51 vector was added on 6-well (or $5^{E}+11$vg on 12-well). Cells were incubated with AAV vectors in differentiation medium for 9 days and were then harvested for RNA and protein analysis.

1. Mamchaoui, K, Trollet, C, Bigot, A, Negroni, E, Chaouch, S, Wolff, A, *et al.* (2011). Immortalized pathological human myoblasts: towards a universal tool for the study of neuromuscular disorders. *Skelet Muscle* **1**: 34.
