# Supplementary material for: Long-Term Efficacy of AAV9-U7snRNA-Mediated Exon 51 Skipping in mdx52 Mice
Source: Mol Ther Methods Clin Dev. 2020 May 4;17:1037–47. doi: 10.1016/j.omtm.2020.04.025 (PMC7240049; doi:10.1016/j.omtm.2020.04.025)
Supplement: Document S2. Article plus Supplemental Information [file mmc2.pdf]

Molecular Therapy
Methods & Clinical Development
Original Article

Long-Term Efficacy of AAV9-U7snRNA-Mediated Exon 51 Skipping in *mdx52* Mice

Philippine Aupy,[1,6] Faouzi Zarrouki,[1,2,6] Quentin Sandro,[1] Cécile Gastaldi,[3] Pierre-Olivier Buclez,[4] Kamel Mamchaoui,[5] Luis Garcia,[1,3] Cyrille Vaillend,[2] and Aurélie Goyenvalle[1,3]

[1]Université Paris-Saclay, UVSQ, INSERM, END-ICAP, 78000 Versailles, France; [2]Neuroscience Paris-Saclay Institute (Neuro-PSI), UMR 9197, Université Paris Sud, CNRS, Université Paris Saclay, 91190 Orsay, France; [3]LIA BAHN, Centre Scientifique de Monaco, 98000 Monaco, Monaco; [4]SQY Therapeutics, UVSQ, END-ICAP, 78180 Montigny le Bretonneux, France; [5]Sorbonne Université, INSERM, Institut de Myologie, U974, Centre de Recherche en Myologie, 75013 Paris, France

Gene therapy and antisense approaches hold promise for the treatment of Duchenne muscular dystrophy (DMD). The advantages of both therapeutic strategies can be combined by vectorizing antisense sequences into an adeno-associated virus (AAV) vector. We previously reported the efficacy of AAV-U7 small nuclear RNA (U7snRNA)-mediated exon skipping in the *mdx* mouse, the *dys*$^{-}$/*utr*$^{-}$ mouse, and the golden retriever muscular dystrophy (GRMD) dog model. In this study, we examined the therapeutic potential of an AAV-U7snRNA targeting the human DMD exon 51, which could be applicable to 13% of DMD patients. A single injection of AAV9-U7 exon 51 (U7ex51) induces widespread and sustained levels of exon 51 skipping, leading to significant restoration of dystrophin and improvement of the dystrophic phenotype in the *mdx52* mouse. However, levels of dystrophin re-expression are lower than the skipping levels, in contrast with previously reported results in the *mdx* mouse, suggesting that efficacy of exon skipping may vary depending on the targeted exon. Additionally, while low levels of exon skipping were measured in the brain, the dystrophin protein could not be detected, in line with a lack of improvement of their abnormal behavioral fear response. These results thus confirm the high therapeutic potential of the AAV-mediated exon-skipping approach, yet the apparent discrepancies between exon skipping and protein restoration levels suggest some limitations of this experimental model.

INTRODUCTION

Duchenne muscular dystrophy (DMD) is a neuromuscular disorder that affects 1 out of 5,000 live male births and is caused by mutations in the dystrophin (*DMD*) gene. Most mutations cause a disruption of the reading frame, leading to the absence of the dystrophin protein at the sarcolemma of muscle fibers, thus resulting in progressive muscle weakness. One of the most promising therapeutic strategies for DMD aims to transform an out-of-frame mutation into an in-frame mutation. This would give rise to an internally truncated but still functional protein, as observed in Becker muscular dystrophy (BMD), in which in-frame mutations may lead to a milder phenotype.[1,2] This so-called exon-skipping strategy can be achieved using antisense oligonucleotides (ASOs) that interfere with splicing signals or regulating elements in the exon, thus leading to the skipping of the targeted exon at the precursor (pre-)mRNA level.[3–6] The applicability of ASO-based therapies has now been demonstrated in clinical trials using different chemistries of ASOs targeting the human dystrophin exon 51 in DMD patients. In September 2016, eteplirsen, a phosphorodiamidate morpholino oligomer (PMO) developed by Sarepta Therapeutics, was approved by the US Food and Drug Administration (FDA), although additional trials were requested to establish the clinical benefit.[7] Recently, gene therapy approaches using adeno-associated virus (AAV) encoding microdystrophin genes have also gained increasing attention. Following many encouraging preclinical studies in mice and dogs,[8,9] several clinical trials have been launched to evaluate this strategy in the clinic. In March 2019, 9 months into their phase 1/2 study, Sarepta Therapeutics announced very encouraging results on four DMD patients treated with AAVrh74.MHCK7.microdystrophin, showing approximately 80% dystrophin-positive fibers.[10]

Both exon-skipping and gene therapy approaches face distinct challenges such as poor cellular uptake and rapid clearance from the circulation for ASOs, which therefore require repeated administrations. Alternatively, AAV-mediated gene therapy is limited by the vector packaging capacity, imposing a much shorter dystrophin (microdystrophin) than the potential dystrophin resulting from exon skipping.

Advantages of these two therapeutic approaches can successfully be combined through the use of AAV-U7 small nuclear RNA (U7snRNA): antisense sequences can be delivered to cells using viral vectors carrying a gene, such as the modified *U7snRNA* gene, from which the antisense sequence is transcribed.[11] U7snRNA is normally involved in histone pre-mRNA 3′ end processing, but it can be converted into a versatile tool for splicing modulation following a small change in the binding site for Sm/Sm-like (Lsm) proteins.[12] This strategy protects the antisense sequence from degradation, as the

Received 27 April 2020; accepted 29 April 2020;
https://doi.org/10.1016/j.omtm.2020.04.025.

[6]These authors contributed equally to this work.

Correspondence: Aurélie Goyenvalle, Université Paris-Saclay, UVSQ, INSERM, END-ICAP, 78000 Versailles, France.
E-mail: aurelie.goyenvalle@uvsq.fr

© 2020 The Author(s).
This is an open access article under the CC BY license (http://creativecommons.org/licenses/by/4.0/).

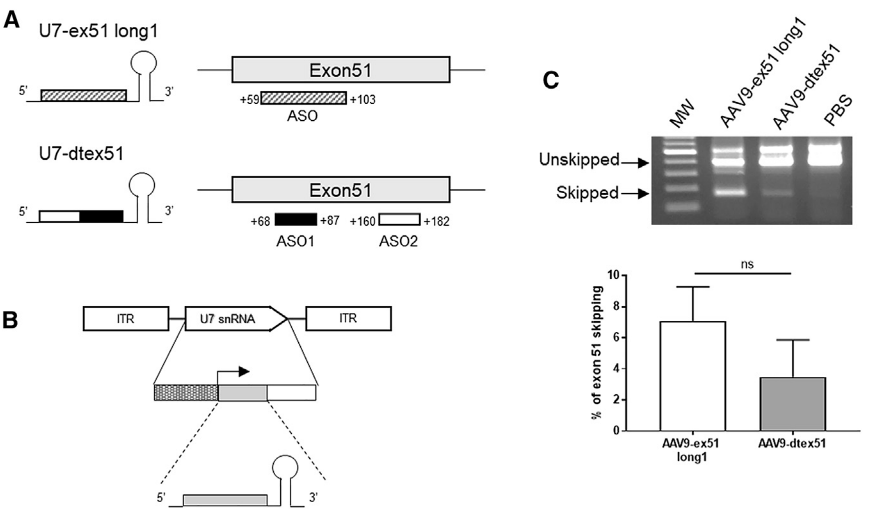

Figure 1. Selection of AAV9-U7snRNA Construct

(A) Representation of the different U7snRNAs and their target on the dystrophin pre-mRNA. (B) Structure of the AAV vectors encoding the different U7snRNA cassettes. The U7snRNA cassette is inserted between the two inverted terminal repeats (ITRs) and is composed of the engineered U7snRNA sequence (gray box) carrying different antisense sequences, placed under the control of its natural U7 promoter (hatched box) and 3′ downstream elements (white box). (C) Two different AAV9-U7snRNA constructs (ex51 long1 and dtex51) were injected intramuscularly in the tibialis anterior (TA) of *mdx52* mice (6E+10 vg/TA). 3 weeks after AAV9-U7 injection, mice were sacrificed and the exon 51 skipping level was evaluated by nested RT-PCR (top) and qRT-PCR (bottom). Results are expressed as mean ± SEM (n = 3 TAs/condition).

ASO is embedded into a small-nuclear ribonucleoprotein particle, and allows its accumulation in the nucleus where the splicing occurs. We have previously demonstrated the therapeutic potential of AAV-mediated exon skipping in mouse models of DMD,[11,13,14] as well as in the golden retriever muscular dystrophy (GRMD) dog model.[15]

Recently, Audentes Therapeutics has announced the expansion of their AAV technology platform to include AAV-mediated exon-skipping approaches. In collaboration with Nationwide Children's Hospital, Audentes is developing AT702, an AAV-U7snRNA designed to induce exon 2 skipping for the treatment of DMD patients with duplications in exon 2 and mutations in exons 1–5 of the dystrophin gene and is expected to commence a phase 1/2 study in 2020.[10]

Considering that 13% of DMD patients are eligible for exon 51 skipping,[16] it is of interest to develop AAV-U7snRNA targeting exon 51 and evaluate its therapeutic potential following systemic delivery in a DMD mouse model. In this study, we evaluated the ability of an AAV9 (AAV serotype 9)-U7 exon 51 (U7ex51) vector to induce exon 51 skipping and to restore expression of the full-length dystrophin (Dp427) in the exon 52-deficient *mdx* mouse model (*mdx52*), in which exon 52 has been deleted by gene targeting, thus causing a rupture of the reading frame and the absence of the protein.[17] In the present study, we show that AAV9-U7ex51 induces widespread and sustained levels of exon 51 skipping, leading to significant restoration of dystrophin and improvement of the dystrophic phenotype after systemic delivery. Considering that AAV9 vectors are able to cross the blood-brain barrier (BBB) when administered intravenously (i.v.),[18] we also investigated the impact of AAV9-U7ex51 on brain dystrophin expression and mouse behavior. While low levels of exon 51 skipping were measured, brain dystrophin expression could not be detected, in line with the lack of behavioral improvement. Altogether these results confirm the therapeutic potential of the AAV-mediated exon-skipping approach but also highlight some limitation of the *mdx52* mouse model.

RESULTS

Validation of the U7snRNA Construct in *mdx52* Mice by Intramuscular Injection

Two different U7snRNA constructs were engineered to target exon 51 of the human dystrophin mRNA: U7-dtex51,[14] containing two antisense sequences targeting internal regions of exon 51, and U7-ex51 long1, containing a single but longer antisense sequence targeting region +59 +103 of exon 51 (Figure 1A; Table 1). Both constructs were shown to efficiently skip the human DMD exon 51 *in vitro* in the human muscle cell line CHQ and *in vivo* following intramuscular injection in the tibialis anterior (TA) muscle of the humanized DMD (hDMD) mouse model (Figures S1A and S1B). In contrast with the hDMD mouse model carrying the entire human DMD gene,[19,20] the *mdx52* mouse model was engineered directly on the murine DMD gene.[17] The antisense sequences of the U7snRNA constructs, designed to target specifically the human DMD exon 51, therefore present a few mismatches with the mouse exon 51: two mismatches out of 45 bases for U7-ex51 long1 (95.5% homology), and four mismatches out of 43 bases for U7-dtex51 (90.7% homology) (Table 1). Thus, it was important to select which of the U7 constructs skips the murine exon 51 most effectively. Both constructs were therefore introduced into distinct AAV9 viral vectors for *in vivo* evaluation (Figure 1B) following intramuscular injections in the TA muscle of *mdx52* mice. Analyses of exon 51 skipping level 3 weeks after AAV9-U7 injection showed a higher potency of the U7-ex51 long1 construct than the U7-dtex51 to skip the murine exon 51 (Figure 1C). These results are consistent with the number of mismatches, since U7-ex51 long1 is the construct with fewer mismatches. We therefore selected the AAV9-ex51 long1 construct for the next *in vivo* evaluation in *mdx52* mice.

AAV9-ex51 Induces Widespread Exon 51 Skipping and Dystrophin Restoration in *mdx52* Mice

mdx52 mice were injected i.v. with 3E+13 vector genomes (vg) of AAV9-ex51 long1 (referred to as AAV9-ex51 hereafter). A first group

Table 1. Antisense Sequences Inserted into U7snRNA Constructs Targeting the Human DMD Exon 51

Name	Target Region in Exon 51	Antisense Sequences
U7-ex51 long1	+59+103	5′-GCAGGTACCTCCAACATCAAGGAAG ATGGCATTTCTAGTTTGGAG-3′
U7-dtex51	(+68+87)/ (+160+182)	5′-CCTCTGTGATTTTATAACTTGAT/TCA AGGAAGATGGCATTTCT-3′

U7-ex51 long1 targets region +59+103 of the human DMD exon 51 and U7-dtex51 targets 2 regions (+68+87 and +160+182). Underlined nucleotides represent the location of mismatches with the mouse exon 51.

of mice was analyzed 8 weeks post-injection to evaluate both exon 51 skipping level and dystrophin protein restoration. The RT-PCR results presented in Figure 2A shows a skipped 51 product in all analyzed tissues. Quantification of exon 51 skipping (Figure 2A, bottom) revealed a significant level of skipping in skeletal muscles (40%–76% of total dystrophin mRNA level) and an average of 18% of skipping across the stomach, duodenum, and ileum smooth muscles. High levels of skipping were particularly found in the heart (90%), which is consistent with the known efficacy of AAV9 in cardiac muscle.[21]

We then analyzed the levels of dystrophin protein re-expression in the different muscles by western blot (Figure 2B). Significant amounts of dystrophin protein were detected in all samples, albeit at relatively lower levels compared with the level of exon 51-skipped mRNA: protein re-expression in skeletal muscles ranged from 10% to 25%, whereas levels of skipping ranged from 40% to 76% in the qRT-PCR analysis. Likewise, only 22% of dystrophin protein was quantified in the heart compared to 90% of exon 51 skipping.

Immunostainings were also performed in muscle sections from the TA, triceps, diaphragm, and heart from wild-type (WT), *mdx52* control mice, and AAV9-ex51-treated mice to determine the localization and distribution of the muscle dystrophin protein (Figure 2C). In WT mice, dystrophin was localized at the sarcolemma of all muscle fibers, whereas dystrophin expression was absent in tissues from *mdx52* mice, except for a few revertant fibers as previously reported.[17] Eight weeks after the i.v. injection of AAV9-ex51, we found about 50%–60% of dystrophin-positive fibers in the different muscles, although most of them expressed a weaker dystrophin signal as compared with WT muscle sections. This was particularly true in the heart, where the intensity of the signal clearly appears lower but where most of the fibers were dystrophin positive.

Interestingly, a difference between skipped mRNA and protein levels was also observed *in vitro* in human DMD myoblasts carrying a similar deletion of exon 52 and transduced with AAV-U7ex51 vector (98% of exon 51 skipping gave rise to 47% of dystrophin restoration, Figure S2).

To evaluate the functional outcome of the restored dystrophin protein, we tested the performance of treated mice in the rotarod test. It has previously been described that *mdx52* mice display lower muscle strength than do WT mice, by measuring their specific force, grip power, and running capacities.[22] In this study, we report that *mdx52* mice have a clear motor deficit in the rotarod test. Indeed, *mdx52* mice had unaffected equilibrium on the immobile rod (session 1) and could walk as well as WT mice when the rod rotated at a low (4 rpm) and constant speed (session 2). However, *mdx52* mice fell off rapidly compared to WT mice when they had to walk on the rotating rod during a progressive acceleration phase (from 4 to 40 rpm in 5 min) in session 3 of this test (fall latency of 116.7 ± 12.09 s in *mdx52* and 196.1 ± 13.47 s in WT mice, $p = 0.0002$), which confirms a significantly reduced muscle strength in *mdx52* mice (Figure 2D). In contrast, AAV9-ex51-treated *mdx52* mice performed as well as the WT mice (fall latency of 199 ± 20.62 s, $p = 0.7427$), demonstrating a significant improvement of muscle function (Figure 2D).

AAV9-ex51 Induces Exon 51 Skipping in the Brain

Considering that AAV9 is known to cross the blood-brain barrier (BBB), albeit in low quantities,[18] we also quantified the level of exon skipping and dystrophin protein expression in brain after AAV9-ex51 injection, more specifically in the cortex, cerebellum, and hippocampus. The RT-PCR results (Figure 3A) reveal the presence of a skipped product in these three brain structures. The qRT-PCR quantification (Figure 3B) detected 4.3%, 4.2%, and 6.7% of exon 51 skipping in the cortex, cerebellum, and hippocampus, respectively, thus confirming the ability of our AAV9 vector to cross the BBB. However, dystrophin protein restoration could not be detected either by western blot (Figure 3C) or immunohistochemistry (Figure S3) in these brain structures.

Moreover, we also investigated the fear response, an emotional response that is known to be drastically enhanced in the classical *mdx* mouse lacking the brain full-length dystrophin due to a nonsense mutation in exon 23.[23,24] This robust phenotype is thought to reflect an enhancement of defensive behavior in response to a threat due to altered function of the fear circuit, independently from motor, respiratory, or cardiac defects.[23,25] This specific phenotype is characterized in mice by measuring the duration of their tonic immobility (freezing) in response to a restraint-induced mild stress. In this study, the mice were submitted to a brief (10-s) restraint and then released in a cage, and their activity was monitored for 5 min. In response to this acute mild stress, the *mdx52* mice spent about 85% of their time freezing, while a baseline immobility level of only 22% was measured in the WT mice (Figure 3D). These results indicate that *mdx52* mice display the same emotional disturbance as do *mdx* mice. However, when this was tested in *mdx52* mice treated with the AAV9-ex51 vector, we did not detect any significant improvement as compared with the control *mdx52* mice, suggesting that the treatment did not have the capacity to compensate for this brain-dependent abnormal behavior (Figure 3D).

Long-Lasting Effect of the AAV9-ex51 Vector

In order to evaluate the long-lasting effect of the AAV9-ex51 treatment, we analyzed a second group of mice 6 months after AAV

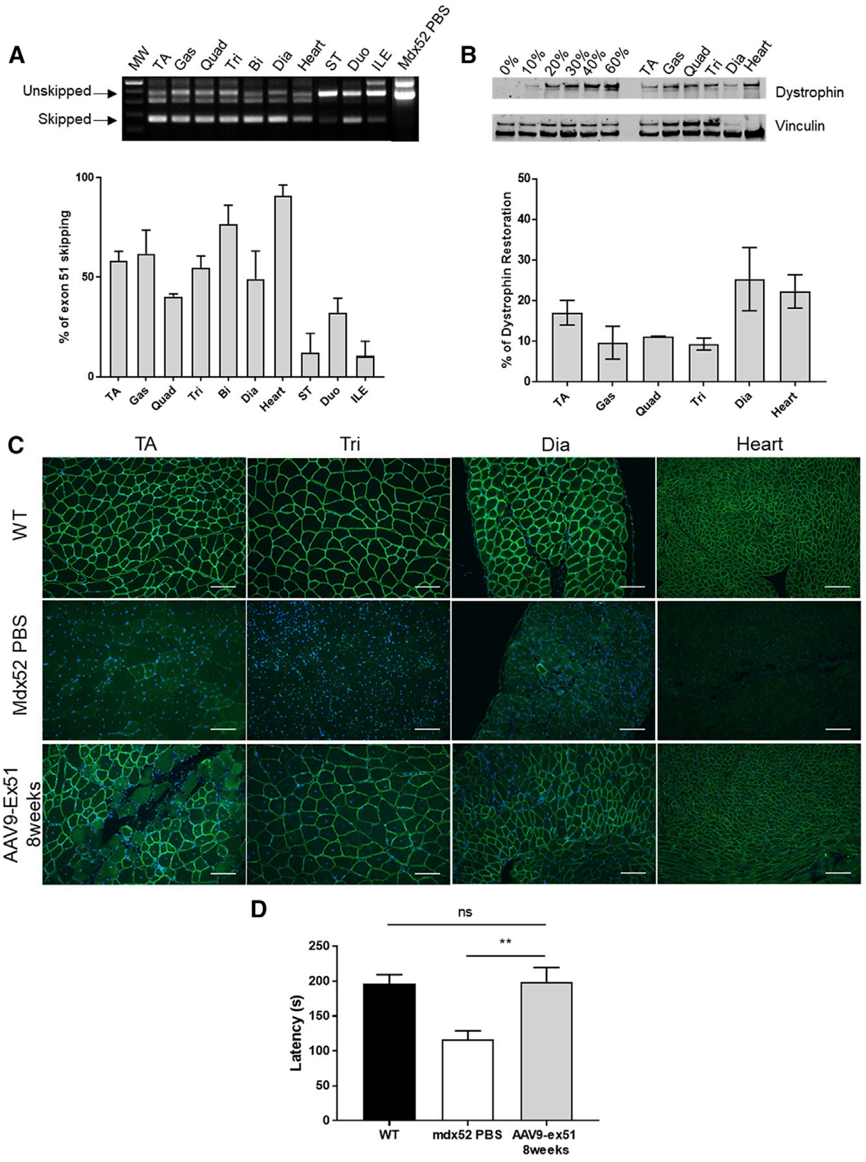

Figure 2. AAV9-ex51 Induces Dystrophin Restoration and Functional Improvement 8 Weeks after Injection

(A) Visualization and quantification of exon 51 skipping level in *mdx52* muscles by nested RT-PCR (top) and qRT-PCR (bottom) (n = 3 mice). (B) Detection of dystrophin restoration in skeletal muscles by western blot compared to control (PBS) *mdx52* mice (0%) and WT mice. 20 μg of total proteins was loaded for all samples, with amounts of WT tissues ranging from 10% to 60% for the WT control. Quantification using Empiria Studio (LI-COR Biosciences) reveals 9%–25% of restored dystrophin across the various muscles (n = 3 mice). (C) Immunostaining of dystrophin in WT, *mdx52* PBS, and AAV9-ex51-treated mice. Scale bars, 100 μm. TA, tibialis anterior; Gas, gastrocnemius; Quad, quadriceps; Tri, triceps; Bi, biceps; Dia, diaphragm; ST, stomach; Duo, duodenum; ILE, ileum. (D) Fall latency during the acceleration phase on the rotating rod (session 3) in the rotarod test (WT, n = 11; *mdx52* PBS, n = 12; AAV9-ex51, n = 5). Results are expressed as mean ± SEM. **p < 0.01 (Mann-Whitney U tests).

injection. The RT-PCR results in Figure 4A demonstrate the presence of the exon 51-skipped product in all analyzed skeletal muscle samples, smooth muscles, and brain structures. qRT-PCR quantification revealed high levels of exon 51 skipping, with an average of about 60% in skeletal muscles, as well as in heart (90%) and smooth muscles (20%). These levels of skipping were not statistically different from those measured 8 weeks after the injection. Low levels of exon 51 skipping were also detected in the brain structures by both RT-PCR and qPCR (Figure 4A). However, again, the brain dystrophin protein was not detectable in these samples (Figure S3) and the enhanced fear phenotype was still not improved by the treatment (Figure S4). In the skeletal muscles and heart, however, dystrophin expression was readily detectable by western blot (Figure 4B) and immunohistochemistry (Figure 4C). Overall dystrophin restoration measured across the different muscles 6 months after the AAV injection was similar (not statistically different) to that observed after 8 weeks, confirming the sustained effect of the treatment.

The functionality of the restored dystrophin was assessed 6 months after the injection using different motor tests. Mice were first placed on an inverted grid for 120 s and their fall latency was recorded (Figure 5A). While WT mice spent the entire time gripped to the grid, *mdx52* mice rapidly fell off (about 2 s after the beginning of the test). After AAV9-ex51 injection, *mdx52* mice performed similarly to WT mice, and a maximal fall latency of 120 s was recorded in all of them (hence the absence of SEM for WT and treated *mdx52* histogram bars). When mice had to grip a wire with their forelimbs (Figure 5B), control *mdx52* mice could not sustain their grip and fell off more rapidly than did WT mice and AAV9-ex51-treated mice, about 2 s after the beginning of the test. In the wire suspension test mice may also express a traction reflex, allowing them to grip the wire with at least one of their hindpaws. This performance was also analyzed using a scoring method as described in Figure S5.[26] *mdx52* mice tried to execute tractions but did not succeed, demonstrating a lack of strength in their forelimbs as reflected by their low score in this analysis. In contrast, both WT and AAV9-ex51-treated *mdx52* mice were able to execute tractions and reached higher scores, as most of them could hang onto the wire with the forepaws and even move along the wire in at least some of the three successive trials, thus demonstrating a strong improvement of muscle strength in treated *mdx52* mice.

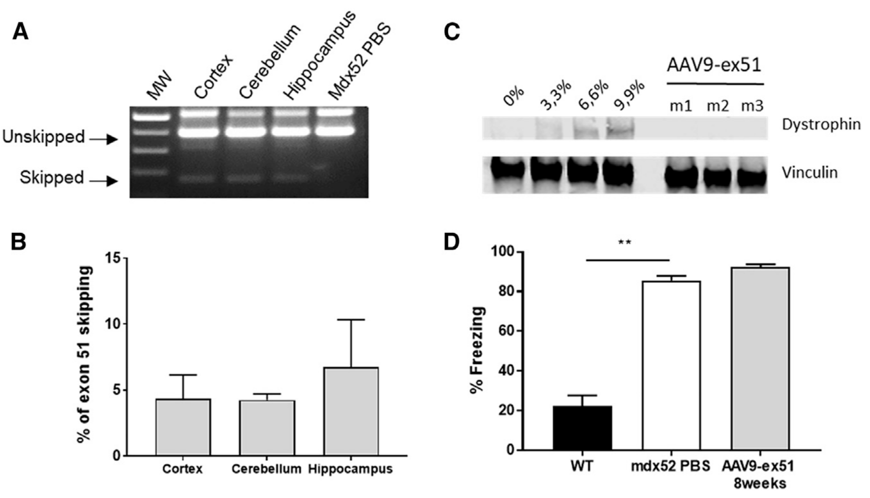

Figure 3. AAV9-ex51 Induces Exon 51 Skipping in the Brain

(A) Visualization of exon 51 skipping in the cortex, cerebellum, and hippocampus of AAV9-Ex51-treated *mdx52* mice compared with *mdx52* PBS mice by nested RT-PCR. (B) Quantification of exon 51 skipping level in *mdx52* mouse brain by qRT-PCR 8 weeks after AAV9-ex51 injection (n = 3 mice). (C) Detection of dystrophin restoration in brain (cerebellum) by western blot compared to *mdx52* mice (0%) and WT mice. 20 μg of total proteins was loaded for all samples, with amounts of WT tissues ranging from 3.3% to 9.9% for the WT control. No dystrophin can be detected in treated mice (n = 3 mice). See the same results for cortex and hippocampus in Figure S2. (D) Freezing response: *mdx52* mice were mildly stressed by a 10-s restraint and then released in a cage to measure their fear response characterized by tonic immobility (freezing behavior) for 5 min. WT mice only showed freezing during about 20% of the 5-min testing period, while freezing amount in control *mdx52* mice reached 80%, thus reflecting their abnormally enhanced fear response. No improvement was observed in *mdx52* mice treated with the AAV9-ex51 vector (WT, n = 5; *mdx52* PBS, n = 6; AAV9-ex51, n = 6). Results are expressed as mean ± SEM. **p < 0.01 (Mann-Whitney U tests).

The treatment effect was also evaluated through the measurement of myomesin-3 (MYOM3) levels in the serum of treated mice. As previously described, MYOM3 is present in sera from DMD patients and some animal models of DMD and is substantially decreased after dystrophin and muscle restoration, making MYOM3 a promising biomarker for monitoring therapeutic outcomes in DMD.[27] Our results confirm that MYOM3 levels are elevated in the serum of control *mdx52* mice and are significantly decreased in AAV9-ex51-treated mice at both time points (8 weeks and 6 months) (Figure 5C). We also confirmed that MYOM3 levels are more reliable than creatine kinase (CK) levels, as exemplified in Figure 5D where levels of CK were only slightly lower 8 weeks and 6 months after AAV9-ex51 treatment but not statistically different from control *mdx52* mice (PBS).

Finally, levels of serum urea, creatinine, albumin, alkaline phosphatase (ALP), bilirubin, and transaminases (alanine aminotransferase [ALT] and aspartate aminotransferase [AST]) were measured 8 weeks and 6 months after AAV9-ex51 injection (Figure 5D), and no significant increase in those levels were observed, suggesting an absence of toxicity of the treatment.

DISCUSSION

In this study, we demonstrate widespread and high levels of exon 51 skipping leading to sustained restoration of muscle dystrophin in the *mdx52* model following a single injection of the AAV9-U7ex51 vector, confirming the therapeutic potential of the AAV-mediated exon skipping approach. *mdx52* mice were injected with a relatively high dose of vector (3E+13 vg of AAV9-ex51) since one of the objectives was actually to reach substantial levels of skipping in order to investigate the resulting levels of protein restoration and potential therapeutic benefit in *mdx52* mice. It cannot be excluded that lower doses of AAV vector encoding a U7-ex51 specific to the mouse exon 51 would induce a similar effect, but the goal here was not to determine the dose/efficiency relationship but rather to evaluate the skipping/protein relationship. Surprisingly, while the levels of exon 51 skipping were high in skeletal muscles (from 40% to 76%) and even higher in heart (90%), the levels of dystrophin protein restoration were lower, ranging between 10% and 25%, which represents on average a 4-fold discrepancy between skipped mRNA and protein levels. In contrast, previous studies in the original *mdx* mouse model carrying a nonsense mutation in exon 23 have shown a rather good correlation between exon 23 skipping and dystrophin restoration, regardless of the skipping approach (ASO or AAV-U7 based).[13,28] In line with our present results, previous work investigating exon 51 skipping induced by ASOs in the *mdx52* model also revealed some discrepancies between exon skipping and dystrophin restoration levels.[22] Aoki et al.[22] even had to use a combination of two ASOs to skip the exon 51 efficiently enough (30%–70%) to allow dystrophin restoration in the range of 10%–30%. In their discussion, the authors commented that the ASO therapeutic dose for exon 51 skipping in the *mdx52* mice (320 mg/kg/dose to reach 20% of dystrophin) was approximately 4-fold higher than doses required for exon 23 skipping in the *mdx* mouse (approximately 80 mg/kg/dose). This highlights a difference between exons, which is important to consider when taking these approaches to the clinic. However, the difference observed between the two models lies more in the protein levels than the skipping levels. The exon 51 skipping levels that we report herein following AAV9-ex51 injection are particularly high and not significantly different from exon 23 skipping levels previously obtained using a similar dose of AAV9-ex23.[13] Nonetheless, this translates into lower levels of dystrophin restoration than those reported after exon 23 skipping.[13] This difference could be due to a defect in protein translation from the exon 51-skipped mRNA or to a difference in DMD mRNA levels between the classical *mdx* and *mdx52* models. DMD transcript imbalance between 5′ and 3′ was previously described and shown to influence dystrophin levels.[29] A detailed

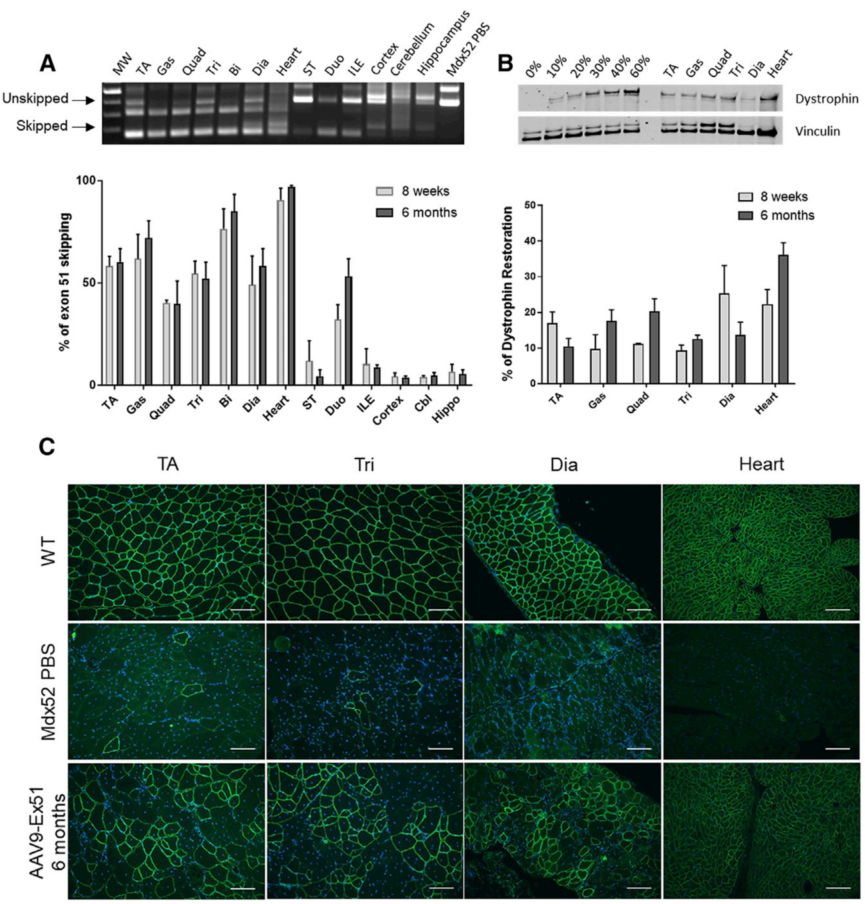

Figure 4. AAV9-ex51 Injection Induces Long-Term Exon 51 Skipping and Dystrophin Restoration

(A) Visualization and quantification of exon 51 skipping level in mice tissues by nested RT-PCR (top) and qRT-PCR (bottom) (n = 3 mice/group). Levels of exon 51 skipping are not significantly different 6 months after AAV9-ex51 long1 injection compared to 8 weeks. (B) Detection of dystrophin restoration in skeletal muscles by western blot compared to *mdx52* mice (0%) and WT mice. 20 μg of total proteins was loaded for all samples, with amounts of WT tissues ranging from 10% to 60% for the WT control. Quantification using Empiria Studio (LI-COR Biosciences reveals no significant changes in percentage of dystrophin restoration between 8 weeks and 6 months (n = 3 mice/group). (C) Immunostaining of dystrophin in WT (top), control *mdx52* (middle), and AAV9-ex51-treated mice (bottom). Scale bars, 100 μm. Results are expressed as mean ± SEM.

study of the 5′-3′ dystrophin transcript imbalance in the *mdx52* would be useful to evaluate whether the slopes are steeper than in the *mdx* mouse model. Alternatively, this discrepancy could be due to an instability of the resulting protein where the Δ51–52 dystrophin may be less stable than the Δ23 dystrophin, and some of it may be degraded or not reaching the membrane to be stabilized within the dystrophin-associated protein complex (DAPC). Interestingly, we also observed this difference *in vitro* in human DMD myotubes carrying a deletion of exon 52, although to a lesser extent.

Several studies have investigated the biophysical properties of internally deleted dystrophin proteins or fragments within the central rod domain, especially around exon 43–51.[30–32] Sahni et al.[31] reported in 2012 that the Δ51–52 fragment had a significantly reduced melting temperature but otherwise no particular stability issues. Subsequent studies conducted using the entire proteins rather than fragments suggested there was little measurable change in stability *in vitro* between exon 51-skipped proteins and full-length human dystrophin.[32]

Considering that we found a less profound difference *in vitro* in human myotubes (∼98% of exon 51 skipping giving rise to approximately 48% of dystrophin protein), it cannot be excluded that the discrepancies may be specific or enhanced in the *mdx52* model due to the particular genetic engineering of the deletion, since exon 52 has been deleted by gene targeting (i.e., replaced with the neomycin gene),[17] as opposed to real deletions at the genomic levels found in DMD patients.

Remarkably, very few patients have been identified with such a specific in-frame deletion of exons 51–52 (only one individual reported in the UMB database with normal CK levels), which could suggest an asymptomatic phenotype in carriers who would therefore never be identified. This would argue in favor of a highly functional Δ51–52 dystrophin, as previously suggested by Aoki et al.,[22] although stability issues cannot be ruled out. In this study, we show that restoration of approximately 20% of Δ51–52 dystrophin allows a strong improvement of muscle and motor functions, as demonstrated by the performance of treated *mdx52* mice in the rotarod, inverted grid, and wire suspension tests, as well as by the decrease of serum MYOM3 levels. This is of particular importance considering that the resulting protein is predicted to lack most of hinge 3 region and that the severity of BMD patients with in-frame deletions including hinge 3 can vary significantly.[33,34] Our functional results therefore confirm previous findings from Aoki et al.[22] suggesting the functionality of Δ51–52 dystrophin.

In this work, we report for the first time an abnormally enhanced defensive behavior in response to a threat in the *mdx52* mouse model, which appears to be fully comparable to the phenotype previously described in the Dp427-deficient *mdx* mouse model. The freezing response is indeed a robust test highlighting a specific neurobehavioral deficit independent of motor, respiratory, or cardiac defects,[24,25] which has been associated with the lack of dystrophin in brain structures involved in emotional processing.[23] We have previously

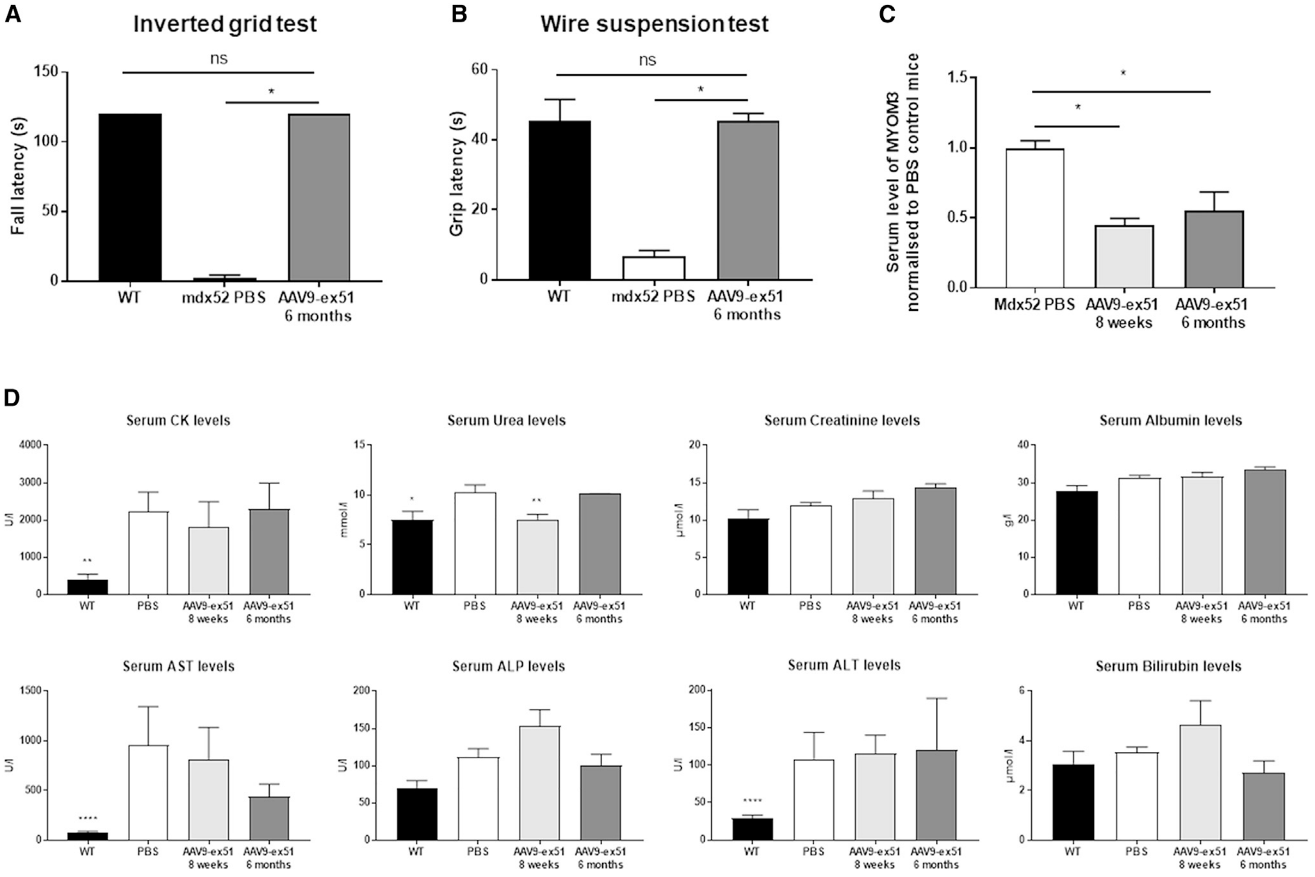

Figure 5. AAV9-Ex51 Induces Long-Term Functional Improvement

(A) Fall latency in the inverted grid test in AAV9-Ex51-treated mice (n = 3), WT mice (n = 4), and control PBS *mdx52* mice (n = 5). A maximum score of 120 s was given when the mice did not fall. (B) Fall latency in the wire suspension test in AAV9-Ex51-treated mice (n = 3), WT mice (n = 4), and control PBS *mdx52* mice (n = 5). (C) Levels of MYOM3 in serum 8 weeks (n = 3) or 6 months (n = 3) after AAV9-ex51 injection as quantified by western blot using Empiria Studio (LI-COR Biosciences) and normalized to control *mdx52* mice (n = 6). (D) Serum CK, urea, creatinine, albumin, AST, ALP, ALT, and bilirubin levels were measured 8 weeks (n = 3) or 6 months (n = 3) after AAV9-ex51 injection compared to WT (n = 11) and control *mdx52* mice (n = 9). Results are expressed as mean ± SEM. *p < 0.05, **p < 0.01, ****p < 0.0001 (Mann-Whitney U tests).

demonstrated that low levels of exon 23 skipping and dystrophin restoration (<6%) in the brain of *mdx* mice following systemic administration of tricyclo-DNA (tcDNA) ASOs result in an apparently full normalization of this fear response.[28,35] Considering the reported ability of AAV9 to cross the BBB at a low level following i.v. administration,[18] we investigated the effect of AAV9-ex51 treatment in the brain. We clearly identified exon 51 skipping in the cortex, hippocampus, and cerebellum of treated *mdx52* mice, at levels that were at least comparable to or even greater than the levels previously reported in the *mdx* mouse with tcDNA ASOs. However, in this study, in treated *mdx52* mice the brain dystrophin protein could not be detected using western blot or immunofluorescence techniques. This may be due to the discrepancy observed in the *mdx52* model between skipping levels and protein restoration (approximately 4-fold). Since the mean level of skipping in the brain samples was about 5%, 4-fold lower levels of dystrophin protein would make it only 1.2% and therefore quite challenging to detect. Consistent with the lack of dystrophin restoration, AAV9-ex51-treated mice did not show any improvement in the behavioral test as compared to the control (untreated) *mdx52* mice. However, we cannot exclude the possibility that a functionally detrimental change of the structure of the brain Δ51–52 dystrophin, such as the partial loss of the hinge 3 domain, could have different effects in muscle and brain tissues. To explore these hypotheses further, we are currently trying to induce higher levels of exon 51 skipping in the CNS using local delivery.

Mouse models lacking multiple dystrophin isoforms, such as the *mdx52* mouse, which also lacks other CNS dystrophins (Dp260, Dp140) in addition to the loss of the full-length Dp427, are expected to present more severe behavioral/cognitive deficits than the *mdx* mouse, which only lacks Dp427. They could therefore represent a particular useful tool to investigate the role of the various dystrophin isoforms in the brain and the potential of currently developed molecular tools for brain gene therapy. However, one should keep in mind that the levels of exon skipping need to be restored to relatively high levels to be able to detect protein restoration in this model.

In this study we also confirm the long-lasting effect of AAV vectors, as a single AAV9-ex51 injection induced sustained levels of exon skipping and dystrophin restoration in skeletal, smooth, and cardiac muscles for up to 6 months. Nevertheless, we have previously reported a progressive loss of both AAV vectors and dystrophin-positive fibers in a 5-year follow-up study in the GRMD model,[15] as well as in the more severe *dys*$^{-}$/*utr*$^{-}$ mouse model.[36] This progressive decay may in part be due to the persistence of the dystrophic process, particularly in severe models, similar to what occurs in BMD. This is of significant importance for future clinical trials since the production of neutralizing antibodies following the first injection of AAV prevents re-administration of this type of vector. The humoral response is also a challenge for patients with pre-existing AAV neutralizing antibodies[37,38] who are usually excluded from AAV trials. Besides the humoral response, the adaptive immune response is often discussed in AAV gene therapy, and the T cell response to AAV capsid has been reported in many trials.[9,39] As an additional hurdle, recent studies suggest that activation of the innate immune response may be a significant concern for high-dose systemic AAV gene therapy.[40,41] These immunological challenges are common to all AAV gene therapy approaches, including the AAV-microdystrophin approach currently in trials. However, the AAV-U7snRNA strategy clearly presents many other advantages, including the time- and tissue-specific effects and avoiding any ectopic expression of the transgene/corrected gene, as opposed to the use of strong promoters in "classical" gene therapy. Moreover, the small size of the U7snRNA gene is particularly convenient, considering the low packaging capacity of AAV vectors, and it even allows the insertion of several snRNA cassettes into a single AAV in order to induce double or even multiple exon skipping.[14] Compared with synthetic ASOs facing poor cellular uptake and rapid circulation clearance, the use of AAV-U7snRNA allows specific subcellular localization of the antisense sequence and long-term correction, and it limits the potential toxicity induced by life-long treatment with ASOs.

In summary despite the immunological barriers faced by viral vectors regarding their potential re-administration, the AAV-U7snRNA-mediated antisense approach represents a very promising tool for the treatment of DMD.

MATERIALS AND METHODS

U7snRNA Constructs and AAV Production

The different U7snRNA constructs specific to the human DMD exon 51 were engineered from the previously described U7smOPT-SD23/BP22 (modified murine *U7snRNA* gene).[11] Antisense sequences targeting mouse exon 23 were replaced by antisense sequences targeting different internal regions of human exon 51. U7-ex51 long1 contains a single 45-mer antisense sequence targeting region +59+103 of exon 51. U7-dtex51 contains 20-mer (+68+87) and 23-mer (+160+182) antisense sequences targeting two internal regions of exon 51. The resulting U7snRNA fragments were then introduced between the inverted terminal repeat (ITR) of a pAAV construct into an AAV vector construct for subsequent AAV vector production. AAV9-pseudotyped vectors were prepared by co-transfection in 293T cells of pAAV-U7, pXX6 encoding adenovirus helper functions and pAAV2/9pITRCO2 that contains the AAV9 rep and cap genes. Vector particles were purified on iodixanol gradients from cell lysates obtained 72 h after transfection, titers were measured by quantitative real-time PCR, and quality control was performed using silver nitrate to check the presence of VP1, VP2, and VP3 proteins, as previously described.[42]

Animal Experiments

Animal procedures were performed in accordance with national and European legislation, approved by the French government (Ministère de l'Enseignement Supérieur et de la Recherche, Autorisation APAFiS #6518). *mdx52* (C57BL/6J) mice were bred in our animal facility at the Plateforme 2Care, UFR des Sciences de la Santé, Université de Versailles-Saint Quentin, and were maintained in a standard 12-h light/12-h dark cycle with free access to food and water. Mice were weaned after 4–5 postnatal weeks, and two to five individuals were housed per cage.

Intramuscular Injections. Three 6- to 8-week-old *mdx52* mice were injected once in the TA, under general anesthesia using 1.5%–2% isoflurane, with 6E+10 vg of AAV9-U7-ex51 long1 or AAV9-U7-dtex51 (n = 3 TA/condition). An age-matched *mdx52* group receiving an equivalent volume of sterile saline was included as control. Animals were euthanized 3 weeks after AAV9 injection, and TAs were harvested and snap-frozen in liquid nitrogen-cooled isopentane and stored at −80°C before further analysis.

i.v. Injections. Six 6- to 8-week-old *mdx52* mice were injected once i.v. in the retro-orbital sinus, under general anesthesia using 1.5%–2% isoflurane, with 3E+13 vg of AAV9-U7-ex51-long1. An age-matched *mdx52* group receiving an equivalent volume of sterile saline was included as control. Blood samples were collected 8 weeks or 6 months after the injection. Animals were euthanized 8 weeks or 6 months after the AAV9 injection, and tissues were harvested and snap-frozen in liquid nitrogen-cooled isopentane and stored at −80°C before further analysis. Sample sizes and n values are indicated in each figure legend. Investigators were blinded for RNA and protein analyses.

Serum Analysis

Analyses of serum CK, ALT, AST, ALP, bilirubin, creatinine, urea, and albumin levels were performed by the pathology laboratory at the Mary Lyon Centre, Medical Research Council, Harwell, Oxfordshire, UK.

For MYOM3 detection, mouse sera were diluted at 1:20 before loading onto 3%–8% Criterion XT Tris-acetate protein gel, following the manufacturer's instructions (Bio-Rad, France). MYOM3 protein was detected by probing the nitrocellulose membrane with MYOM3 primary rabbit polyclonal antibody (Proteintech, Manchester, UK), followed by incubation with a goat anti-rabbit secondary antibody (IRDye 800CW goat anti-rabbit immunoglobulin G [IgG], LI-COR Biosciences, Germany). Bands were visualized using the Odyssey imaging system (LI-COR Biosciences, Lincoln, NE USA). Signal

intensities in treated samples were quantified and normalized to PBS control mice signals using the Image Studio software (LI-COR Biosciences, Germany).

RNA Analysis

Total RNA was isolated from muscle cells or intervening muscle sections collected during cryosection using TRIzol reagent according to the manufacturer's instructions (Thermo Fisher Scientific, USA). Aliquots of 500 ng of total RNA were used for RT-PCR analysis using the Access RT-PCR system (Promega, USA) in a 50-μL reaction using the external primers exon 49 forward (5′-GATTGAAGTAACAGTT CACGG-3′) and exon 53 reverse (5′-CCAGCCATTGTGTTGAA TCC-3′). The cDNA synthesis was carried out at 45°C for 45 min, directly followed by the primary PCR of 30 cycles of 95°C (30 s), 58°C (1 min), and 72°C (2 min). 2 μL of these reactions was then re-amplified in nested PCRs by 30 cycles of 95°C (30 s), 58°C (1 min), and 72°C (1 min) using the internal primers exon 50 forward (5′-TTTACTTCGGGAGCTGAGGA-3′) and the same exon 53 reverse (5′-CCAGCCATTGTGTTGAATCC-3′). PCR products were analyzed on 2% agarose gels. Exon 51 skipping was also measured by TaqMan qRT-PCR as previously described using TaqMan assays that were designed against the exon 50–51 or exon 50–53 templates using a custom assay design tool (Life Technologies) (assay Ex50–51: Mm01216958_m1 and assay Ex50–53: AID1U27). 50 ng of cDNA was used as input per reaction, and all assays were carried out in triplicate. Assays were performed under fast cycling conditions on a Bio-Rad CFX384 Touch real-time PCR detection system, and all data were analyzed using the absolute copy number method. For a given sample the copy number of exon 50–51 and exon 50–52 were determined using the standards Ex48–55Δ52 and Ex48–55Δ51+52, respectively. Exon 51 skipping was then expressed as a percentage of total dystrophin (calculated by the addition of exon 50–51 and exon 50–53 copy numbers).

Western Blot Analysis

Protein extracts were obtained from pooled muscle sections treated with radioimmunoprecipitation assay (RIPA) lysis and extraction buffer (Thermo Fisher Scientific, USA) complemented with SDS powder (5% final) (Bio-Rad, France), and the total protein concentration was determined with a bicinchoninic acid (BCA) protein assay kit (Thermo Fisher Scientific, USA). Samples were denatured at 100°C for 3 min, and 20 μg of protein was loaded onto NuPAGE 3%–8% Tris-acetate protein gels (Invitrogen), following the manufacturer's instructions. Dystrophin protein was detected by probing the membrane with NCL-DYS1 primary monoclonal antibody (Novocastra, Newcastle, UK), and vinculin was detected as an internal control with the hVin-1 primary antibody (Sigma), followed by incubation with a goat anti-mouse secondary antibody (IRDye 800CW goat anti-mouse IgG, LI-COR Biosciences, Germany). Bands were visualized using the Odyssey CLx system (LI-COR Biosciences, Germany). The quantification was done using a standard curve with 0%, 10%, 20%, 30%, 40%, and 60% of the corresponding WT tissues and normalized to an internal control (vinculin) (Empiria Studio, LI-COR Biosciences, Germany).

Immunohistochemistry Analysis

Sections of 10 μm were cut from at least two-thirds of the muscle length of the various tissues (TA, triceps brachialis, diaphragm, and cardiac muscle) at 100-μm intervals. Cryosections were examined for dystrophin expression using the rabbit polyclonal antibody dystrophin (dilution 1:500; catalog no. RB-9024-P Thermo Scientific), which was then detected by goat anti-rabbit IgG Alexa Fluor 488 (dilution 1:500; Thermo Scientific). Controls prepared by omitting primary antibody showed no specific staining. Images were taken at equivalent locations and exposure times using a Leica DMI-4000 microscope (Leica Microsystems, ×20 objective) equipped with a Zyla 5.5 sCMOS camera (Oxford Instruments Group). Images were cropped, and scale bar of 100 μm was added using ImageJ software.

Brain fresh-frozen cryosections of 30 μm were collected onto Superfrost+ slides, thawed for 2 min at room temperature (RT), fixed in acetone/methanol (1:1) for 5 min at −20°C, washed in PBS, incubated first in a blocking solution for 45 min (10% normal goat serum, 0.3% Triton X-100, and 1% BSA), then overnight at 4°C with a monoclonal anti-dystrophin primary antibody (DYS1 Leica; dilution, neat), and washed and incubated with secondary antibody Alexa Fluor 647 (1:400, 1 h, RT). Controls prepared by omitting the primary antibody showed no specific staining. Images were taken at equivalent locations and exposure times using a laser scanning confocal microscope (Zeiss LSM 700, ×40 objective). Stacks of seven to eight images (1,024 × 1,024 pixels) spaced by 1 μm were recorded at a magnification of 156 nm/pixel.

Functional Evaluation

Freezing Response

The mouse was restrained by a trained experimenter by grasping the scruff and back skin between the thumb and index finger while securing the tail between the third and little fingers and tilting the animal upside down. After 10 s, the mouse was released to a new cage (16 × 28 cm, with 12-cm-high walls; illumination, 100 lx) and then videotracked for 5 min using the ANY-maze software (Stoelting). All mice were tested between 10:00 a.m. and 1:00 p.m. Unconditioned fear responses induced by this acute stress were characterized by periods of tonic immobility (freezing) during the 5-min recording period in the novel cage. Complete immobilization of the mouse, except for respiration, was regarded as a freezing response. This was typically quantified as episodes of immobility lasting at least 1 s, with a 90% immobility sensitivity (10% body motion allowed). In all experiments, the percent time the mouse was freezing was calculated for group comparisons. The investigator was blinded to the mouse group assignment during experiments.

Rotarod

Locomotor function was evaluated by using a mouse rotarod with adjustable speed and accelerating mode (catalog no. #47600, Ugo Basile, Italy). Three to five mice were tested in parallel; the apparatus was cleaned with 100% ethanol after each trial. On the first day between 9:00 a.m. to 1:00 p.m. (session 1), equilibrium was tested by placing each mouse on the non-rotating rod, with its body axis perpendicular

to the rod longitudinal axis. The time the animal stayed on the rod was recorded and the trial stopped when the animal fell or after 180 s. In the afternoon between 1:00 p.m. and 5:00 p.m. (session 2), mice were placed on the rod rotating at a constant speed (4 rpm), its head directed against the direction of rotation so that it had to progress forward and synchronize its walk with the speed of the rod to maintain balance. Training consisted of five successive trials with a 15-min inter-trial interval (ITI); fall latency was recorded during each trial with a 180-s cutoff duration. Locomotor function was analyzed 24 h later (session 3) by placing mice on the rotating rod, which accelerated from 4 to 40 rpm in a 5-min trial. Evaluation then consisted of four successive trials with a 60-min ITI; fall latency was recorded during each trial with a 300-s cutoff duration, and a mean score was then calculated.

Inverted Grid Test

Mice were placed individually on a cage wire screen about 35 cm above a table. After slowly inverting the screen upside down to 180° the ability to maintain a grip was monitored (fall latency) and a maximum score of 120 s given if the animal did not fall. Testing was repeated three times with a 5-min ITI and the mean fall latency was then calculated.

Wire Suspension Test

The mouse was hung by the forepaws on a 25-cm wire (3 mm in diameter) resting on two vertical supports and elevated 35 cm above a flat surface. Three trials spaced by a 5-min pause were performed with each trial limited to a 60-s duration. The amount of time spent by the mouse hanging on to the wire and the latency to touch the wire with one hindpaw were recorded during each trial; a mean score was then calculated. Other qualitative parameters were recorded and a score was attributed corresponding to the best performance achieved within the minute of testing according to the following scale:[43] 0, fell off; 1, clung to the wire with two forepaws; 2, attempted to climb on to the wire besides clinging to it with two forepaws; 3, hung on to the wire with two forepaws and one or both hind paws; 4, hung on to the wire with all four paws with the tail additionally wrapped around the bar; 5, escaped to one of the supports.

Statistical Analysis

Data were expressed as means ± SEM and analyzed with the GraphPad Prism 7 software (GraphPad, San Diego, CA, USA). The "n" refers to the number of mice per group. Comparisons of statistical significance were assessed by non-parametric Mann-Whitney U tests for two-group comparisons and a Kruskal-Wallis test for comparison of three or more groups followed by a Dunn's multiple comparison test. Significant levels were set at $^{*}p < 0.05$, $^{**}p < 0.01$, $^{***}p < 0.001$, and $^{****}p < 0.0001$.

SUPPLEMENTAL INFORMATION

Supplemental Information can be found online at https://doi.org/10.1016/j.omtm.2020.04.025.

AUTHOR CONTRIBUTIONS

P.A., F.Z., and A.G. performed the *in vivo* experiments. Q.S. and P.-O.B. produced the AAV vectors. C.G. performed the muscle immunostainings and MYOM3 western blot. F.Z. analyzed the CNS. K.M. immortalized the human myoblasts. P.A., A.G., and C.V. wrote the manuscript. A.G., C.V., and L.G. conceived the project and supervised the entire study.

CONFLICTS OF INTEREST

P.-O.B. is an employee of SQY Therapeutics, and L.G. is consulting for Audentes Therapeutics. The remaining authors declare no competing interests.

ACKNOWLEDGMENTS

We thank the platform for the immortalization of human cells from the Institut de Myologie for the human-derived cells. We thank Jun Tanihata and Shin'ichi Takeda from the National Center of Neurology and Psychiatry for providing *mdx52* mice. This work was supported by the Agence Nationale de la Recherche (Chair of Excellence HandiMedEx); the Institut National de la Santé et de la Recherche Médicale (INSERM); the Centre National de la Recherche Scientifique (CNRS, France); the University Paris-Sud (France); the Association Monegasque contre les Myopathies (AMM); the Duchenne Parent Project France (DPPF); and the Fondation UVSQ. P.A. was supported by the Fondation pour la Recherche Médicale (FRM grant PLP20161036676), and F.Z. was supported by the Association Française contre les Myopathies (AFM).

REFERENCES

1. Koenig, M., Beggs, A.H., Moyer, M., Scherpf, S., Heindrich, K., Bettecken, T., Meng, G., Müller, C.R., Lindlöf, M., Kaariainen, H., et al. (1989). The molecular basis for Duchenne versus Becker muscular dystrophy: correlation of severity with type of deletion. Am. J. Hum. Genet. *45*, 498–506.
2. Monaco, A.P., Bertelson, C.J., Liechti-Gallati, S., Moser, H., and Kunkel, L.M. (1988). An explanation for the phenotypic differences between patients bearing partial deletions of the DMD locus. Genomics *2*, 90–95.
3. Pramono, Z.A., Takeshima, Y., Alimsardjono, H., Ishii, A., Takeda, S., and Matsuo, M. (1996). Induction of exon skipping of the dystrophin transcript in lymphoblastoid cells by transfecting an antisense oligodeoxynucleotide complementary to an exon recognition sequence. Biochem. Biophys. Res. Commun. *226*, 445–449.
4. Dunckley, M.G., Manoharan, M., Villiet, P., Eperon, I.C., and Dickson, G. (1998). Modification of splicing in the dystrophin gene in cultured *Mdx* muscle cells by antisense oligoribonucleotides. Hum. Mol. Genet. *7*, 1083–1090.
5. Mann, C.J., Honeyman, K., Cheng, A.J., Ly, T., Lloyd, F., Fletcher, S., Morgan, J.E., Partridge, T.A., and Wilton, S.D. (2001). Antisense-induced exon skipping and synthesis of dystrophin in the *mdx* mouse. Proc. Natl. Acad. Sci. USA *98*, 42–47.
6. Aartsma-Rus, A., Janson, A.A., Heemskerk, J.A., De Winter, C.L., Van Ommen, G.J., and Van Deutekom, J.C. (2006). Therapeutic modulation of DMD splicing by blocking exonic splicing enhancer sites with antisense oligonucleotides. Ann. N Y Acad. Sci. *1082*, 74–76.
7. Echevarría, L., Aupy, P., and Goyenvalle, A. (2018). Exon-skipping advances for Duchenne muscular dystrophy. Hum. Mol. Genet. *27* (R2), R163–R172.
8. Le Guiner, C., Servais, L., Montus, M., Larcher, T., Fraysse, B., Moullec, S., Allais, M., François, V., Dutilleul, M., Malerba, A., et al. (2017). Long-term microdystrophin gene therapy is effective in a canine model of Duchenne muscular dystrophy. Nat. Commun. *8*, 16105.
9. Duan, D. (2018). Systemic AAV micro-dystrophin gene therapy for duchenne muscular dystrophy. Mol. Ther. *26*, 2337–2356.
10. Crudele, J.M., and Chamberlain, J.S. (2019). AAV-based gene therapies for the muscular dystrophies. Hum. Mol. Genet. *28* (R1), R102–R107.

11. Goyenvalle, A., Vulin, A., Fougerousse, F., Leturcq, F., Kaplan, J.-C., Garcia, L., and Danos, O. (2004). Rescue of dystrophic muscle through U7 snRNA-mediated exon skipping. Science *306*, 1796–1799.

12. Schümperli, D., and Pillai, R.S. (2004). The special Sm core structure of the U7 snRNP: far-reaching significance of a small nuclear ribonucleoprotein. Cell. Mol. Life Sci. *61*, 2560–2570.

13. Goyenvalle, A., Babbs, A., Wright, J., Wilkins, V., Powell, D., Garcia, L., and Davies, K.E. (2012). Rescue of severely affected dystrophin/utrophin-deficient mice through scAAV-U7snRNA-mediated exon skipping. Hum. Mol. Genet. *21*, 2559–2571.

14. Goyenvalle, A., Wright, J., Babbs, A., Wilkins, V., Garcia, L., and Davies, K.E. (2012). Engineering multiple U7snRNA constructs to induce single and multiexon-skipping for Duchenne muscular dystrophy. Mol. Ther. *20*, 1212–1221.

15. Vulin, A., Barthélémy, I., Goyenvalle, A., Thibaud, J.L., Beley, C., Griffith, G., Benchaouir, R., le Hir, M., Unterfinger, Y., Lorain, S., et al. (2012). Muscle function recovery in golden retriever muscular dystrophy after AAV1-U7 exon skipping. Mol. Ther. *20*, 2120–2133.

16. Aartsma-Rus, A., Fokkema, I., Verschuuren, J., Ginjaar, I., van Deutekom, J., van Ommen, G.J., and den Dunnen, J.T. (2009). Theoretic applicability of antisense-mediated exon skipping for Duchenne muscular dystrophy mutations. Hum. Mutat. *30*, 293–299.

17. Araki, E., Nakamura, K., Nakao, K., Kameya, S., Kobayashi, O., Nonaka, I., Kobayashi, T., and Katsuki, M. (1997). Targeted disruption of exon 52 in the mouse dystrophin gene induced muscle degeneration similar to that observed in Duchenne muscular dystrophy. Biochem. Biophys. Res. Commun. *238*, 492–497.

18. Foust, K.D., Nurre, E., Montgomery, C.L., Hernandez, A., Chan, C.M., and Kaspar, B.K. (2009). Intravascular AAV9 preferentially targets neonatal neurons and adult astrocytes. Nat. Biotechnol. *27*, 59–65.

19. Bremmer-Bout, M., Aartsma-Rus, A., de Meijer, E.J., Kaman, W.E., Janson, A.A., Vossen, R.H., van Ommen, G.J., den Dunnen, J.T., and van Deutekom, J.C. (2004). Targeted exon skipping in transgenic hDMD mice: A model for direct preclinical screening of human-specific antisense oligonucleotides. Mol. Ther. *10*, 232–240.

20. 't Hoen, P.A., de Meijer, E.J., Boer, J.M., Vossen, R.H., Turk, R., Maatman, R.G., Davies, K.E., van Ommen, G.J., van Deutekom, J.C., and den Dunnen, J.T. (2008). Generation and characterization of transgenic mice with the full-length human DMD gene. J. Biol. Chem. *283*, 5899–5907.

21. Zincarelli, C., Soltys, S., Rengo, G., Koch, W.J., and Rabinowitz, J.E. (2010). Comparative cardiac gene delivery of adeno-associated virus serotypes 1–9 reveals that AAV6 mediates the most efficient transduction in mouse heart. Clin. Transl. Sci. *3*, 81–89.

22. Aoki, Y., Nakamura, A., Yokota, T., Saito, T., Okazawa, H., Nagata, T., and Takeda, S. (2010). In-frame dystrophin following exon 51-skipping improves muscle pathology and function in the exon 52-deficient *mdx* mouse. Mol. Ther. *18*, 1995–2005.

23. Sekiguchi, M., Zushida, K., Yoshida, M., Maekawa, M., Kamichi, S., Yoshida, M., Sahara, Y., Yuasa, S., Takeda, S., and Wada, K. (2009). A deficit of brain dystrophin impairs specific amygdala GABAergic transmission and enhances defensive behaviour in mice. Brain *132*, 124–135.

24. Vaillend, C., and Chaussenot, R. (2017). Relationships linking emotional, motor, cognitive and GABAergic dysfunctions in dystrophin-deficient *mdx* mice. Hum. Mol. Genet. *26*, 1041–1055.

25. Yamamoto, K., Yamada, D., Kabuta, T., Takahashi, A., Wada, K., and Sekiguchi, M. (2010). Reduction of abnormal behavioral response to brief restraint by information from other mice in dystrophin-deficient *mdx* mice. Neuromuscul. Disord. *20*, 505–511.

26. Helleringer, R., Le Verger, D., Li, X., Izabelle, C., Chaussenot, R., Belmaati-Cherkaoui, M., Dammak, R., Decottignies, P., Daniel, H., Galante, M., and Vaillend, C. (2018). Cerebellar synapse properties and cerebellum-dependent motor and non-motor performance in *Dp71*-null mice. Dis. Model. Mech. *11*, dmm033258.

27. Rouillon, J., Poupiot, J., Zocevic, A., Amor, F., Léger, T., Garcia, C., Camadro, J.M., Wong, B., Pinilla, R., Cosette, J., et al. (2015). Serum proteomic profiling reveals fragments of MYOM3 as potential biomarkers for monitoring the outcome of therapeutic interventions in muscular dystrophies. Hum. Mol. Genet. *24*, 4916–4932.

28. Relizani, K., Griffith, G., Echevarría, L., Zarrouki, F., Facchinetti, P., Vaillend, C., Leumann, C., Garcia, L., and Goyenvalle, A. (2017). Efficacy and safety profile of tricyclo-DNA antisense oligonucleotides in Duchenne muscular dystrophy mouse model. Mol. Ther. Nucleic Acids *8*, 144–157.

29. Spitali, P., van den Bergen, J.C., Verhaart, I.E.C., Wokke, B., Janson, A.A.M., van den Eijnde, R., den Dunnen, J.T., Laros, J.F., Verschuuren, J.J., 't Hoen, P.A., and Aartsma-Rus, A. (2013). DMD transcript imbalance determines dystrophin levels. FASEB J. *27*, 4909–4916.

30. Ruszczak, C., Mirza, A., and Menhart, N. (2009). Differential stabilities of alternative exon-skipped rod motifs of dystrophin. Biochim. Biophys. Acta *1794*, 921–928.

31. Sahni, N., Mangat, K., Le Rumeur, E., and Menhart, N. (2012). Exon edited dystrophin rods in the hinge 3 region. Biochim. Biophys. Acta *1824*, 1080–1089.

32. McCourt, J.L., Rhett, K.K., Jaeger, M.A., Belanto, J.J., Talsness, D.M., and Ervasti, J.M. (2015). In vitro stability of therapeutically relevant, internally truncated dystrophins. Skelet. Muscle *5*, 13.

33. Beggs, A.H., Hoffman, E.P., Snyder, J.R., Arahata, K., Specht, L., Shapiro, F., Angelini, C., Sugita, H., and Kunkel, L.M. (1991). Exploring the molecular basis for variability among patients with Becker muscular dystrophy: dystrophin gene and protein studies. Am. J. Hum. Genet. *49*, 54–67.

34. Carsana, A., Frisso, G., Tremolaterra, M.R., Lanzillo, R., Vitale, D.F., Santoro, L., and Salvatore, F. (2005). Analysis of dystrophin gene deletions indicates that the hinge III region of the protein correlates with disease severity. Ann. Hum. Genet. *69*, 253–259.

35. Goyenvalle, A., Griffith, G., Babbs, A., El Andaloussi, S., Ezzat, K., Avril, A., Dugovic, B., Chaussenot, R., Ferry, A., Voit, T., et al. (2015). Functional correction in mouse models of muscular dystrophy using exon-skipping tricyclo-DNA oligomers. Nat. Med. *21*, 270–275.

36. Le Hir, M., Goyenvalle, A., Peccate, C., Précigout, G., Davies, K.E., Voit, T., Garcia, L., and Lorain, S. (2013). AAV genome loss from dystrophic mouse muscles during AAV-U7 snRNA-mediated exon-skipping therapy. Mol. Ther. *21*, 1551–1558.

37. Calcedo, R., and Wilson, J.M. (2013). Humoral immune response to AAV. Front. Immunol. *4*, 341.

38. Louis Jeune, V., Joergensen, J.A., Hajjar, R.J., and Weber, T. (2013). Pre-existing anti-adeno-associated virus antibodies as a challenge in AAV gene therapy. Hum. Gene Ther. Methods *24*, 59–67.

39. Mingozzi, F., and High, K.A. (2017). Overcoming the host immune response to adeno-associated virus gene delivery vectors: the race between clearance, tolerance, neutralization, and escape. Annu. Rev. Virol. *4*, 511–534.

40. Zaiss, A.K., Cotter, M.J., White, L.R., Clark, S.A., Wong, N.C.W., Holers, V.M., Bartlett, J.S., and Muruve, D.A. (2008). Complement is an essential component of the immune response to adeno-associated virus vectors. J. Virol. *82*, 2727–2740.

41. Hinderer, C., Katz, N., Buza, E.L., Dyer, C., Goode, T., Bell, P., Richman, L.K., and Wilson, J.M. (2018). Severe toxicity in nonhuman primates and piglets following high-dose intravenous administration of an adeno-associated virus vector expressing human SMN. Hum. Gene Ther. *29*, 285–298.

42. Dias Florencio, G., Precigout, G., Beley, C., Buclez, P.-O., Garcia, L., and Benchaouir, R. (2015). Simple downstream process based on detergent treatment improves yield and in vivo transduction efficacy of adeno-associated virus vectors. Mol. Ther. Methods Clin. Dev. *2*, 15024.

43. Aruga, J., Ogura, H., Shutoh, F., Ogawa, M., Franke, B., Nagao, S., and Mikoshiba, K. (2004). Locomotor and oculomotor impairment associated with cerebellar dysgenesis in Zic3-deficient (Bent tail) mutant mice. Eur. J. Neurosci. *20*, 2159–2167.

OMTM, Volume *17*

Supplemental Information

Long-Term Efficacy of AAV9-U7snRNA-Mediated Exon 51 Skipping in *mdx52* Mice

Philippine Aupy, Faouzi Zarrouki, Quentin Sandro, Cécile Gastaldi, Pierre-Olivier Buclez, Kamel Mamchaoui, Luis Garcia, Cyrille Vaillend, and Aurélie Goyenvalle

Supplemental information includes:

-5 supplementary figures with figure legends

-supplementary methods related to the supplementary figures

SUPPLEMENTARY FIGURES

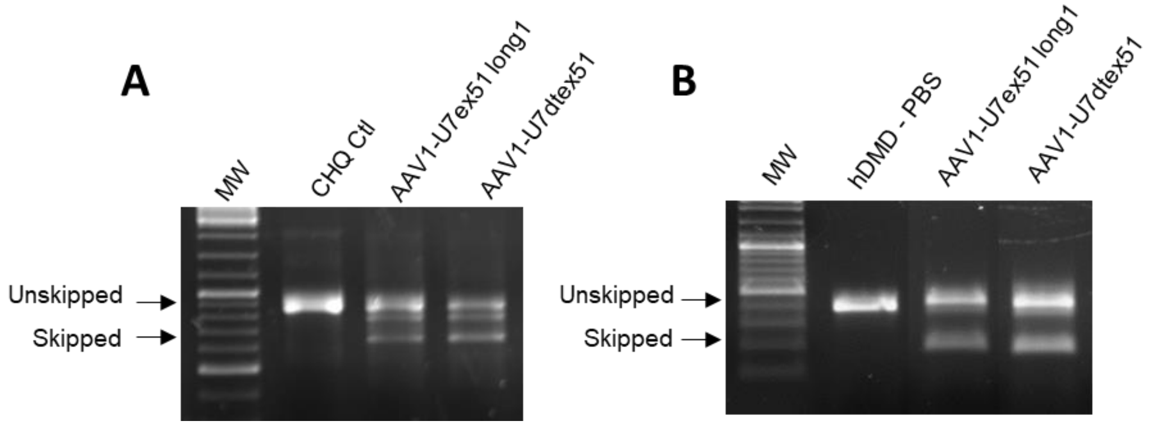

<u>Supplementary Figure 1</u>**: Both U7ex51 constructs efficiently skip the human exon 51** ***in vitro*** **and** ***in vivo*****.** The two different U7snRNA constructs (ex51 long1 and dtex51) were introduced into AAV1 vectors for preliminary evaluation. A) detection of exon 51 skipping in human immortalized skeletal cell line CHQ transduced with 5^{E}+11vg of AAV1 vector encoding the various U7ex51. B) Detection of exon 51 skipping in the TA of hDMD mice injected intramuscularly with 1^{E}+11vg. TA muscles were analyzed 3 weeks after AAV1-U7 injection.

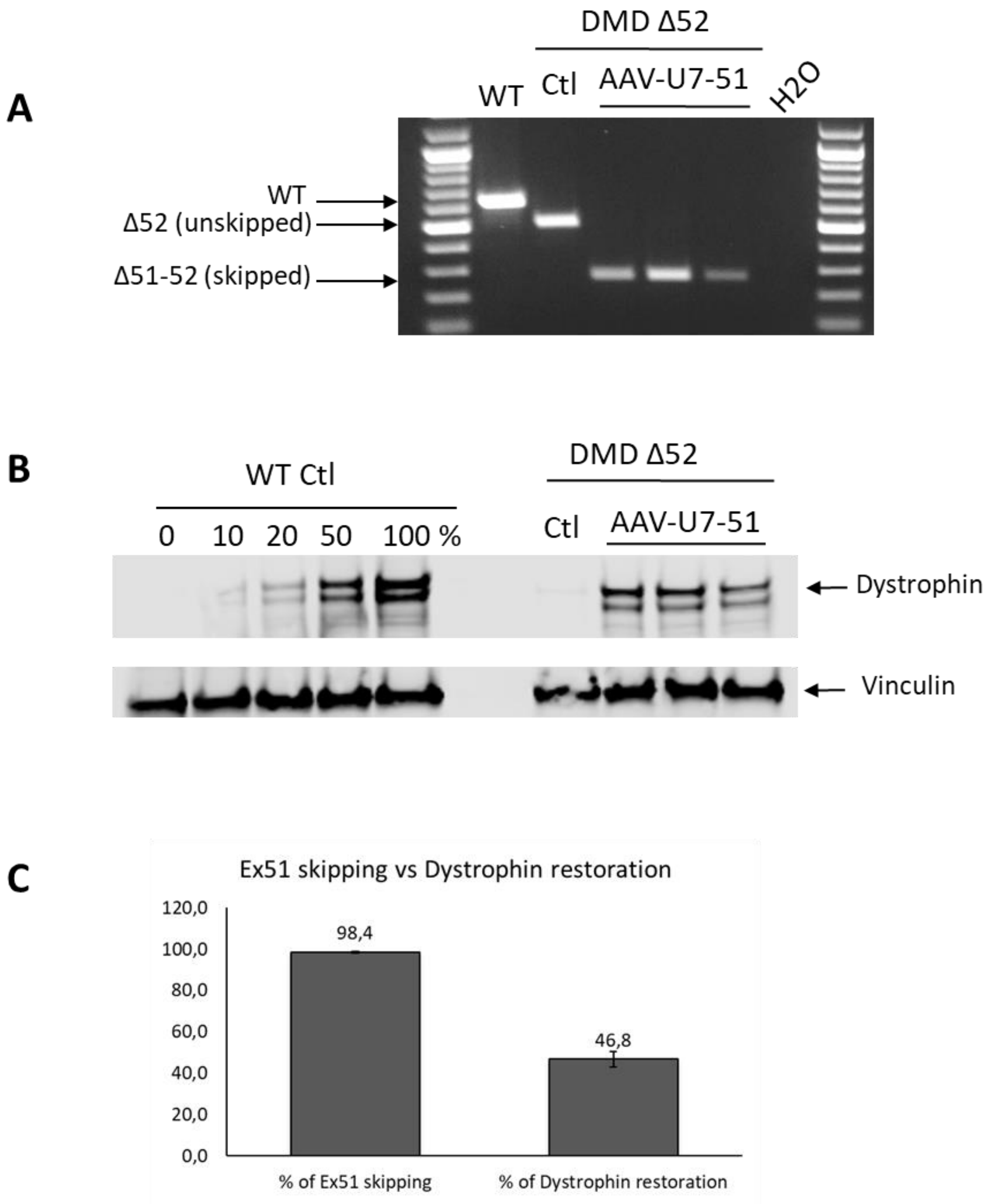

Supplementary Figure 2: Comparison between exon 51 skipping and dystrophin protein restoration *in vitro*. A) Detection of exon 51 skipping in human immortalised DMD Δ52 (KM571) myotubes transduced with AAV1-U7ex51. B) Detection of dystrophin protein in human immortalised DMD Δ52 (KM571) myotubes transduced with AAV1-U7ex51. A standard curve made of WT protein lysate (CHQ myotubes) mixed with DMD Δ52 (KM571) myotubes protein lysate was used to quantify the level of dystrophin restoration. C) Graph presenting the quantification of skipping and dystrophin protein levels. N=3 transduction experiments.

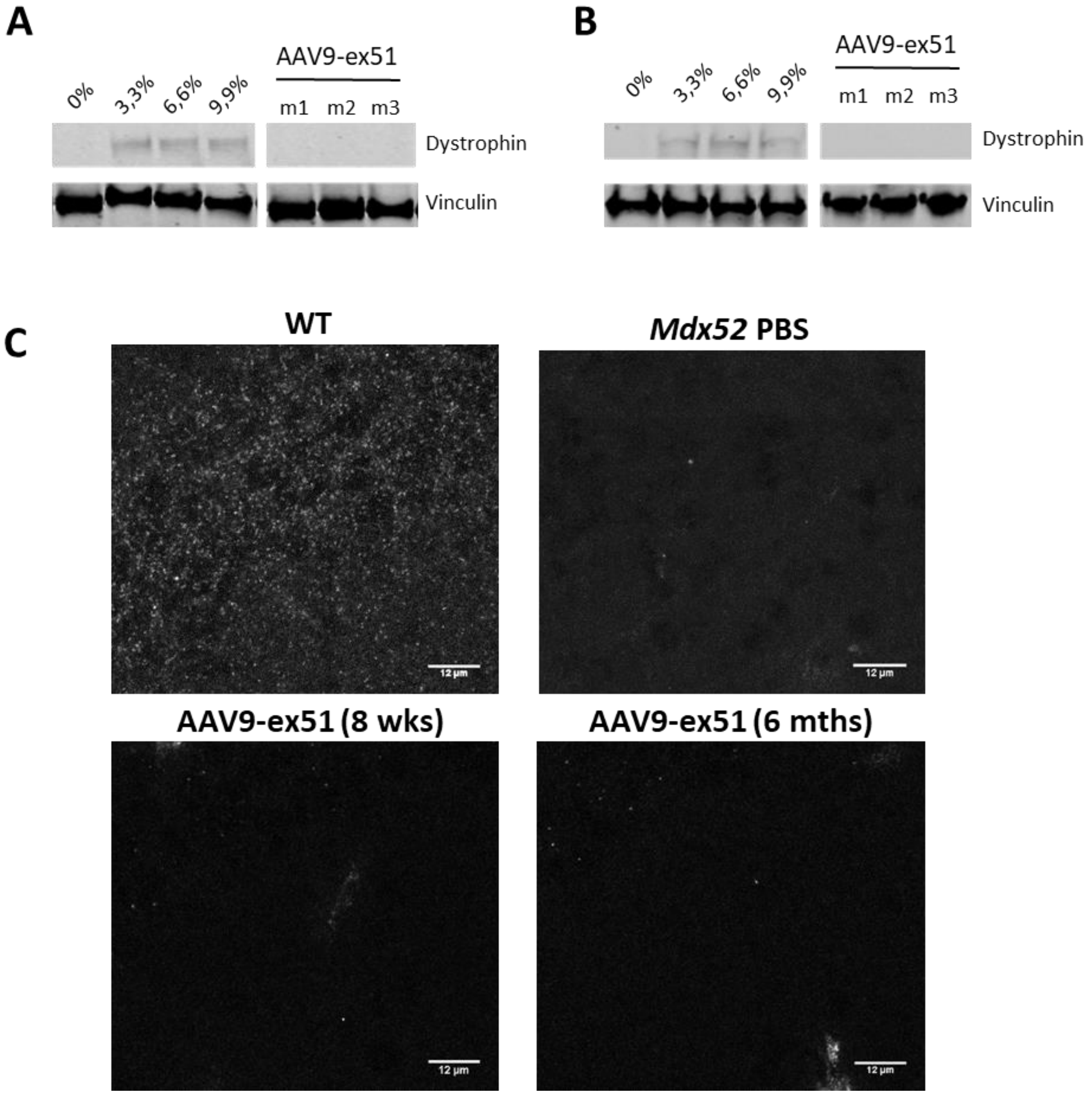

<u>Supplementary Figure 3</u>: **AAV9-ex51 intravenous injection does not restore Dp427 in the brain.** Detection of dystrophin restoration in A) Hippocampus and B) Cortex by western blot compared to control *mdx52* mice (0%) and WT mice. 20µg of total proteins were loaded for all samples, with amounts of WT tissues ranging from 3,3% to 9,9% for the WT control. No dystrophin could be detected in treated mice (n=3 mice). C) Immunostaining of dystrophin in hippocampus (stratum pyramidale) of WT, control *mdx52-PBS and* AAV9-ex51 treated mice, 8 weeks or 6 months after the intravenous injection. Scale bar, 12µm.

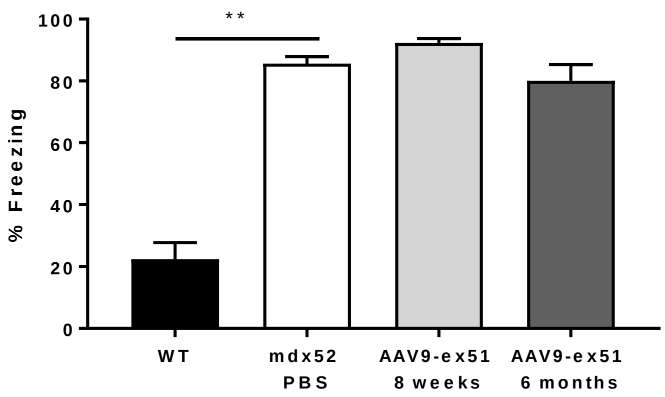

<u>Supplementary Figure 4</u>**: AAV9-ex51 has no effect on *mdx52* freezing response 8 weeks or 6 months post-injection.** Mice were restrained for a brief (10s) period and released to a cage to analyze their fear response. Freezing duration was measured for 5 min. WT mice showed freezing during 20% of the 5-min testing period, while *mdx52* mice expressed a freezing amount of 80%. No improvement was observed after AAV9-ex51 injection. (WT n=5, *mdx52* PBS n=6, AAV9-ex51 8weeks n=6, AAV9-ex51 6months n=3). Results are expressed as means ± SEM. **p<0,01 (Mann-Whitney U tests).

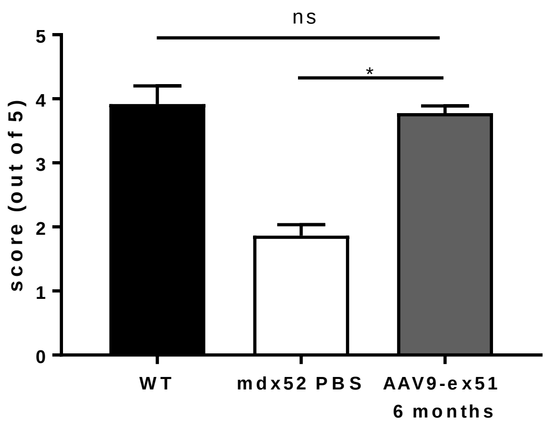

Supplementary figure 5: AAV9-ex51 improves traction capacity in *mdx52* mice. During the wire suspension test, a score was given to determine the mouse capacity to execute tractions. 0= fall directly, 1= grip the wire with forepaws and wait, 2= grip and try traction, 3= successful traction reflex (wire touched by at least one hindpaw), 4= successful traction reflex with all four paws and tail additionally wrapped around the wire, 5= successful traction reflex, tail around the wire and walk along the wire to escape to one of the supports. WT n=4, *mdx52* PBS n=5, AAV9-ex51 n=3. Results are expressed as mean ± SEM. *p<0,05 (Mann-Whitney U tests).

SUPPLEMENTARY MATERIALS AND METHODS

Cells and AAV transduction

Immortalized human skeletal muscle cells derived from a healthy subject (CHQ) and a DMD patient harboring a deletion of exon 52 (ID KM571) in the DMD gene were generated by transduction with human telomerase-expressing and cyclin dependent kinase 4-expressing vectors in the Institute of Myology human cell immortalization platform, as previously described [1]. Cells were cultured in proliferation conditions with Skeletal Muscle Cell Growth Medium (Promocell), supplemented with the skeletal muscle supplement mix (Promocell), 20% fetal bovine serum (Life Technologies), and antibiotics (50 U penicillin and 50 mg/ml streptomycin; Life Technologies). For AAV transduction, 65,000 or 130,000 cells were seeded in 12-well or 6-well culture plates respectively (depending on further analysis required, RNA only or RNA and WB analysis). Two days after seeding, at approximately 80% confluence, medium was replaced with differentiation medium (DMEM with 2% horse serum (GE Healthcare), insulin-transferrin solution (Sigma) and antibiotics) and 1^{E}+12vg of AAV1-U7ex51 vector was added on 6-well (or 5^{E}+11vg on 12-well). Cells were incubated with AAV vectors in differentiation medium for 9 days and were then harvested for RNA and protein analysis.

1. Mamchaoui, K, Trollet, C, Bigot, A, Negroni, E, Chaouch, S, Wolff, A, *et al.* (2011). Immortalized pathological human myoblasts: towards a universal tool for the study of neuromuscular disorders. *Skelet Muscle* **1**: 34.
